# Simplified quantification of [^18^F]FE-PE2I PET in Parkinson’s disease: Discriminative power, test–retest reliability and longitudinal validity during early peak and late pseudo-equilibrium

**DOI:** 10.1177/0271678X20958755

**Published:** 2020-09-21

**Authors:** Joachim Brumberg, Vera Kerstens, Zsolt Cselényi, Per Svenningsson, Mathias Sundgren, Patrik Fazio, Andrea Varrone

**Affiliations:** 1 Centre for Psychiatry Research, Department of Clinical Neuroscience, Karolinska Institutet & Stockholm Health Care Services, Stockholm, Sweden; 2 Department of Nuclear Medicine, University Hospital Würzburg, Würzburg, Germany; 3 AstraZeneca Translational Science Centre at Karolinska Institutet PET CoE, Stockholm, Sweden; 4 Department of Clinical Neuroscience, Section Neuro, Karolinska Institutet, Stockholm, Sweden; 5 Department of Neurology, Karolinska University Hospital, Stockholm, Sweden

**Keywords:** Dopamine transporter, [^18^F]FE-PE2I, Parkinson’s disease, reliability, specific binding ratio

## Abstract

Quantification of dopamine transporter (DAT) availability with [^18^F]FE-PE2I PET enables the detection of presynaptic dopamine deficiency and provides a potential progression marker for Parkinson`s disease (PD). Simplified quantification is feasible, but the time window of short acquisition protocols may have a substantial impact on the reliability of striatal binding estimates. Dynamic [^18^F]FE-PE2I PET data of cross-sectional (33 PD patients, 24 controls), test–retest (9 patients), and longitudinal (12 patients) cohorts were used to assess the variability and reliability of specific binding ratios (SBR) measured during early peak and late pseudo-equilibrium. Receiver operating characteristics area under the curve (PD vs. controls) was high for early (0.996) and late (0.991) SBR. Early SBR provided more favourable effect size, absolute variability, and standard error of measurement than late SBR (caudate: 1.29 vs. 1.23; 6.9% vs. 9.8%; 0.09 vs. 0.20; putamen: 1.75 vs. 1.67; 7.7% vs. 14.0%; 0.08 vs. 0.17). The annual percentage change was comparable for both time windows (−7.2%–8.5%), but decline was significant only for early SBR. Whereas early and late [^18^F]FE-PE2I PET acquisitions have similar discriminative power to separate PD patients and controls, the early peak equilibrium acquisition can be recommended if [^18^F]FE-PE2I is used to measure longitudinal changes of DAT availability.

## Introduction

The pathophysiology of Parkinson`s disease (PD) is characterized by the loss of dopaminergic cell bodies in the substantia nigra^1,2^ and the degeneration of nigrostriatal projections.^3^ The impairment of dopamine-related neurotransmission in the striatum is linked to most of the classical motor features (i.e. tremor, rigidity, and bradykinesia) of early PD.^4^ A key molecule for dopaminergic function is the dopamine transporter (DAT), which is localized on the plasma membrane of presynaptic cell bodies, axons and nerve terminals and removes free dopamine from the synaptic cleft.^5^

Molecular imaging of the DAT enables the quantification of the presynaptic neuronal integrity and thereby allows to assess the dopaminergic depletion in patients with regard to their clinical presentation.^6^ At present, the main indication for DAT imaging in clinical use is to support differential diagnosis in patients with suspected neurodegenerative parkinsonism by distinguishing PD from essential tremor.^7,8^ In the last years, DAT quantification got increasingly attention for the use as biomarker in the evaluation of potential disease-modifying treatments and neuroprotective agents in clinical trials.^9^ In this context, DAT imaging serves as a secondary outcome measure for treatment effects^10,11^ and has recently qualified as enrichment biomarker.^12^

Several radioligands targeting the DAT have been developed over the last decades, but only few SPECT ligands have reached a broad clinical application.^13^ To take advantage of the higher resolution and sensitivity of modern PET systems, the novel radioligand [^18^F]-(*E*)-*N*-(3-iodoprop-2-enyl)-2β-carbofluoroethoxy-3β-(4´-methylphenyl)nortropane ([^18^F]-FE-PE2I) was recently evaluated in humans.^14^ [^18^F]-FE-PE2I has good affinity (*K*_i_ = 12 nM) and selectivity to the DAT,^15^ shows appropriate kinetics and favourable metabolism in non-human primates,^16^ and a similar metabolism in PD patients and healthy controls.^17^ Furthermore, non-invasive quantification methods, such as the simplified reference tissue model (SRTM) and the Logan graphical analysis,^17,18^ provide accurate estimates of DAT binding potential (*BP*_ND_).

However, these quantification methods rely on dynamic PET acquisitions over 90 min, which is not always compatible with a clinical setting, where scan time might be limited or patients cannot endure a long scan duration. Previous studies examined to what extent the calculation of the specific binding ratio (SBR) is feasible for [^18^F]-FE-PE2I PET.^19–21^ SBR is a simplified quantification method based on static images of a reduced scan time. Besides scan duration, the optimal time window for SBR estimation according to the [^18^F]-FE-PE2I time activity curves is of particular interest: whereas first results proposed a static acquisition during the radioligands early peak equilibrium,^19^ also image data of the late pseudo-equilibrium have been recently used^20^ and recommended as more favourable for SBR estimation.^21^

This study aims to expand on earlier SBR findings with a larger cohort of PD patients and control subjects and with two subgroups of PD patients, who underwent two [^18^F]-FE-PE2I PET examinations to assess either test–retest reliability or longitudinal changes after a two-year follow-up. The purpose was to understand whether simplified quantification of DAT is more reliable during the early peak or the late pseudo-equilibrium, and if a reduction of scan time to 18 min is feasible during early and late equilibrium. The time window that provides more reliable quantitative estimates should be preferably used for two potential clinical indications of [^18^F]-FE-PE2I PET: differential diagnosis of parkinsonism and measurement of disease progression in PD.

## Material and methods

### Subjects

The participants included in this study were part of three studies approved by the Ethics Committee of the Stockholm Region, by the Swedish Ethical Review Authority, by the Radiation Safety Committee of the Karolinska University Hospital, Stockholm, Sweden, and by the Swedish Medicinal Product Agency. The studies were registered as Clinical Trials in the EudraCT database (2011-002005-30, 2017-001585-19, and 2017-003327-29). The studies were conducted according to the ethical standards of the Ethics Committee of the Stockholm Region and the Swedish Ethical Review Authority, and were in line with the Helsinki Declaration of 1975 (and as revised in 1983). Written, informed consent was obtained from all subjects prior to participation. Healthy controls were recruited by an advertisement in a local newspaper. PD patients were contacted at the Movement Disorder Clinic of the Karolinska University Hospital, Stockholm, Sweden, the Academic Specialist Center, Stockholm, Sweden, and through the Swedish Parkinson patient`s association in Stockholm. All participants underwent the same screening procedure, i.e. exclusion of clinically relevant comorbidities, psychiatric conditions, illicit drug abuse or alcoholism, as assessed by structured interview, physical examination, blood tests, electrocardiogram, and brain MRI. Mini-Mental State Examination was performed to exclude cognitive decline. PD patients fulfilled the clinical diagnosis of PD according to the UK Parkinson Disease Brain Bank criteria.^22^ In total, 24 healthy subjects (62 ± 8 years) and 33 PD patients (cross-sectional cohort, 63 ± 9 years) were included. Demographic and clinical data are presented in Table 1 and in the Supplemental Data.

**Table 1.** Demographic and clinical data of controls and PD patient cohorts.

	Sex	MMSE	Disease duration	H&Y
Controls	8 f/16 m	29.3 ± 0.7	n/a	n/a
PD cross-sectional	10 f/23 m	29.0 ± 1.1	4.0 ± 3.3	1.5
PD test–retest	3 f/6 m	29.4 ± 1.0	6.7 ± 3.5	1.5
PD longitudinal	3 f/9 m	28.8 ± 1.0	2.6 ± 3.2	1.4

Note: Data are presented as mean ± standard deviation.

MMSE: Mini-Mental State Examination; H&Y: Hoehn and Yahr stage; f: female; m: male.

### Imaging procedures

All participants underwent brain MRI scans on a 3 Tesla system (Discovery MR750; GE Healthcare) prior to PET examination as part of the initial evaluation and to delineate anatomic brain volumes of interests (VOI). Two subgroups of patients were invited to perform a second [^18^F]-FE-PE2I PET examination with the purpose to investigate test–retest reliability (10 PD patients) or longitudinal DAT binding changes (20 PD patients). The final numbers of patients, who had two [^18^F]-FE-PE2I PET examinations of sufficient quality for data analysis were reduced due to various reasons, such as technical failure during the scan, loss of contact with the patient, or inability/unwillingness to participate in a second PET examination at follow-up. They underwent PET scans either within 12 ± 8 days (test–retest cohort: 9 PD patients, 65 ± 7 years) or after 2 ± 0 years (longitudinal cohort: 12 PD patients, 62 ± 8 years). All patients performed PET measurements after suspension of dopaminergic replacement therapies for at least 12 h. [^18^F]-FE-PE2I was prepared via nucleophilic radiofluorination of its tosylate precursor as previously described.^23^ Details of molar activity, injected radioactivity and injected mass are provided in the Supplemental Material.

Dynamic PET measurements were obtained using a high-resolution research tomograph (HRRT) system (Siemens Medical Solutions). A 6-min transmission scan with a ^137^Cs source was performed for attenuation correction. [^18^F]-FE-PE2I was injected as i.v. bolus over 10 s, and the catheter was flushed with 10 mL NaCl. Emission data were acquired in list mode over 93 min. PET data were reconstructed in 37 frames of increasing duration (8 × 10 s, 5 × 20 s, 4 × 30 s, 4 × 60 s, 4 × 180 s, 12 × 360 s) using three-dimensional ordinary Poisson ordered subset expectation maximization with modelling of the system`s point spread function. Frame-to-frame motion correction of reconstructed images was applied as previously described.^24^

### Image analysis and DAT quantification

Image processing and analysis were performed using an in-house pipeline named Solena written in MATLAB (MATLAB r2014b, The MathWorks, Inc.). Within Solena, T1-weighted MP-RAGE sequences of each individual were segmented with FreeSurfer (FreeSurfer v6.0.0, http://surfer.nmr.mgh.harvard.edu/)^25^ and the generated segmentation masks were used to define VOIs of the caudate nucleus and the putamen, and one reference region containing the cerebellum. Subsequently, MRI and dynamic PET images were co-registered. Different outcome measures were used to quantify DAT density in each VOI: dynamic PET data were analysed with simplified reference tissue model (SRTM)^26^ to estimate binding potential (*BP*_ND_), which was considered the reference standard.^14,17,18^ Specific binding ratio (SBR) as clinical outcome measure for [^18^F]-FE-PE2I was calculated during early peak and late pseudo-equilibrium using 30 min static acquisitions as well as shorter acquisitions of 18 min. Starting time points and duration of the investigated time windows were chosen according to previous findings.^19,20^ The following four windows were used: static images between 15 and 45 min (frame 24–29; early SBR), between 27 and 45 min (frame 27–29; short early SBR), between 51 and 81 min (frame 31–35; late SBR), and between 57 and 75 min (frame 32–34; short late SBR) were created by averaging the corresponding time frames in the original dynamic images. SBR was calculated as SBR =SUV_VOI_/SUV_CER_ – 1. Early and late SBR values for 30 min and 18 min windows were calculated also for images with lower resolution as previously described^19^ (see Supplemental Material).

### Statistical analysis

All analyses were performed using the statistics software R (R v3.6.1, http://www.R-project.org/).

#### Cross-sectional cohort and control subjects

Linear regression analysis and *r*^2^ were used to assess correlations between *BP*_ND_ and early and late SBR measures. The bias, defined as a measure of the percentage difference between *BP*_ND_ and SBR was calculated using the following formula

$$\mathrm{Bias}=100\times \frac{\bar{\mathrm{SBR}}-\bar{{BP}_{ND}}}{\bar{{BP}_{ND}}}$$

The coefficient of variation (COV) was used as a measure of variability and obtained by dividing the standard deviation (σ) by the mean (µ) of each outcome measure

$$\mathrm{COV}=\frac{\sigma }{\mu }$$

Furthermore, Cohen`s effect size *d* was estimated to assess the ability of *BP*_ND_ and SBR to differentiate PD patients and healthy controls

$$\mathrm{Cohen}`s\;d=\;\frac{\mu_{\mathrm{controls}}-\mu_{PD\;\mathrm{patients}}}{\mathrm{pooled}\;\sigma }$$

Lastly, we evaluated group differences with a two-sample *t* test (*p* < 0.05 with Bonferroni correction for two VOIs) and performed a receiver operating characteristics (ROC) analysis (PD patients vs. controls) to calculate the area under the curve (AUC) for *BP*_ND_ and SBR in the putamen.

#### Test–retest cohort

Test–retest data were used to evaluate the agreement and reliability of outcome measures.^27^ The absolute variability (AbsVar) refers to the agreement between the two measurements and was calculated for *BP*_ND_, early SBR measures, and late SBR measures:

$$\mathrm{AbsVar}=\frac{\left|{|\mathrm{SBR}}^{\mathrm{PET}2}-{\mathrm{SBR}}^{\mathrm{PET}1}|\right|}{\frac{1}{2}\left({\mathrm{SBR}}^{\mathrm{PET}1}+{\mathrm{SBR}}^{\mathrm{PET}2}\right)}$$

Intraclass correlation coefficient (ICC) was assessed for all binding estimates as a measure of reliability and differentiability and was calculated according to a one-way random model.^28^ We also calculated the standard error of measurement (SEM), which indicates the precision of the individual’s binding estimate and is expressed by the standard error of each measurement around the estimated binding value.^29^ *σ* in the following formula refers to both measurements of each individual

$$\mathrm{SEM}=\sigma \times \sqrt{1-\mathrm{ICC}}$$

#### Longitudinal cohort

Group differences of binding estimates between baseline and two-year follow-up PET measurements were evaluated by using the paired *t* test (*p* < 0.05 with Bonferroni correction for two VOIs). To assess binding differences in each VOI and individual, the annual percentage rate of change (APC) was calculated for *BP*_ND_, early SBR measures, and late SBR measures according to the formula

$$\mathrm{APC}=\left(\frac{{\mathrm{SBR}}^{\mathrm{PET}2}-{\mathrm{SBR}}^{\mathrm{PET}1}}{{\mathrm{SBR}}^{\mathrm{PET}1}}\times 100\right)/{\mathrm{years}}^{\mathrm{PET}2-\mathrm{PET}1}$$

## Results

### Linear regression analysis and dispersion metrics

**Figure 1.**
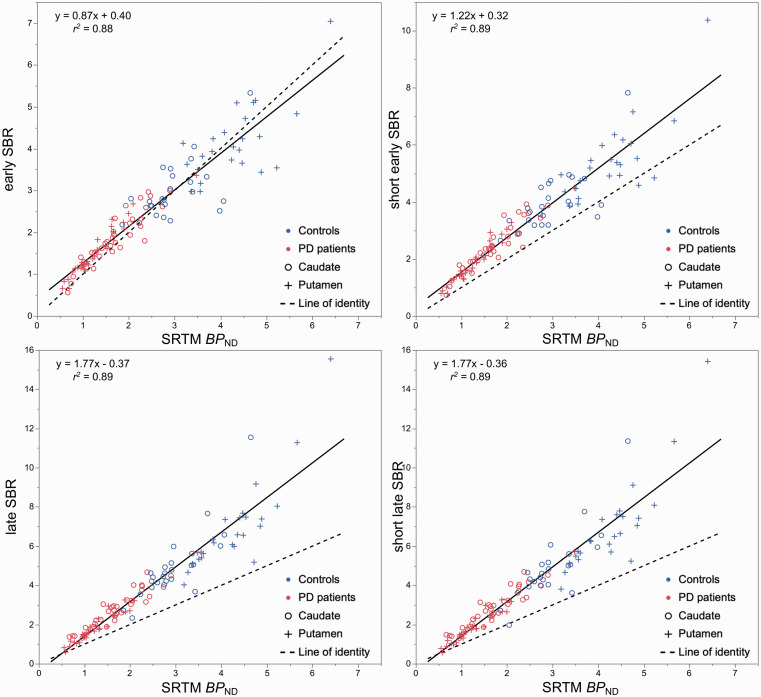
Scatter plots and linear regression analysis between specific binding ratio (SBR) during early peak (left) and late pseudo-equilibrium (right), and binding potential (*BP*_ND_) estimated with the simplified reference tissue model (SRTM).

Regression analysis showed that early (*r*^2^ = 0.88, *p* < 0.001; short early SBR: *r*^2^ = 0.89, *p* < 0.001) and late SBR (*r*^2^ = 0.89, *p* < 0.001; late early SBR: *r*^2^ = 0.89, *p* < 0.001) were highly correlated with *BP*_ND_ (Figure 1). Early SBR values in caudate and putamen were close to *BP*_ND_ in control subjects and showed a slight (caudate) to moderate (putamen) overestimation in PD patients. Short early SBR moderately overestimated *BP*_ND_ in caudate and putamen in both groups. Late SBR values overestimated *BP*_ND_ by ∼50% or more in both regions and groups, and were in close agreement to short late SBR (Table 2). In both groups, the variability of outcome measures was lowest and close to *BP*_ND_ for early SBR and highest for late SBR in both regions (Table 3).

**Table 2.** Binding estimates and bias in controls and PD patients.

BindingEstimates	Controls		PD patients	
	Caudate	Putamen	Caudate	Putamen
SRTM *BP*_ND_	2.98 ± 0.66	4.30 ± 0.79	1.73 ± 0.58	1.28 ± 0.56
Early SBR	2.99 ± 0.70	4.15 ± 0.88	1.90 ± 0.63	1.51 ± 0.61
Short early SBR	4.00 ± 1.04	5.45 ± 1.36	2.47 ± 0.81	1.85 ± 0.81
Late SBR	4.97 ± 1.82	6.96 ± 2.39	2.84 ± 0.94	1.89 ± 0.96
Short late SBR	4.95 ± 1.82	6.96 ± 2.39	2.86 ± 0.94	1.89 ± 0.97
	Controls		PD patients	
Bias (%)	Caudate	Putamen	Caudate	Putamen
Early SBR	0.4	−3.4	9.6	18.6
Short early SBR	34.0	27.0	42.4	45.3
Late SBR	66.7	62.1	63.9	48.4
Short late SBR	65.9	62.1	65.1	48.2

Note: Data are presented as mean ± standard deviation.

SRTM: simplified reference tissue model; *BP*_ND_: binding potential; SBR: specific binding ratio.

**Table 3.** Effect size, variability, test–retest metrics and longitudinal change of DAT binding measures.

	Cohen`s *d*		COV (%)		AbsVar (%)	
	Caudate	Putamen	Caudate	Putamen	Caudate	Putamen
SRTM *BP*_ND_	1.44	1.84	38.5	64.5	7.4 ± 6.0	6.6 ± 7.6
Early SBR	1.29	1.75	36.1	57.3	6.9 ± 5.2	7.7 ± 7.2
Short early SBR	1.29	1.73	38.1	61.9	7.1 ± 5.2	8.4 ± 8.6
Late SBR	1.23	1.67	46.2	75.6	9.8 ± 8.8	14.0 ± 13.9
Short late SBR	1.21	1.67	45.9	75.6	9.4 ± 9.8	15.2 ± 16.0
	ICC		SEM		APC (%)	
SRTM *BP*_ND_	0.96	0.95	0.10	0.05	−8.3 ± 7.8	−7.3 ± 8.6
Early SBR	0.97	0.85	0.09	0.08	−7.2 ± 7.5	−7.4 ± 8.1
Short early SBR	0.95	0.89	0.15	0.11	−7.6 ± 8.6	−8.0 ± 9.0
Late SBR	0.96	0.91	0.20	0.17	−7.5 ± 10.4	−8.5 ± 12.1
Short late SBR	0.96	0.90	0.19	0.18	−7.8 ± 10.4	−8.3 ± 13.2

Note: Data are presented as mean ± standard deviation.

COV: coefficient of variation; AbsVar: absolute variability; ICC: intraclass correlation coefficient; SEM: standard error of measurement; APC: annual percentage rate of change; SRTM: simplified reference tissue model; *BP*_ND_: binding potential; SBR: specific binding ratio.

### Discriminative analysis between PD patients and controls

Similar effect sizes in the caudate and the putamen were observed for all five DAT binding measures. Highest Cohen`s *d* was observed for *BP*_ND_ and lowest for late SBR (Table 3). Cohen`s *d* of short early and short late SBR were each close to the corresponding 30-min SBR. Two-sample *t* test showed highly significant group differences (*p* < 0.0001) for all measures in both striatal regions (Table 2). Likewise, ROC analysis showed similarly high capability to differentiate PD patients from controls for all three outcome measures in the putamen (*BP*_ND_ AUC: 0.996; early SBR AUC: 0.996; short early SBR AUC: 0.994; late SBR AUC: 0.991; short late SBR AUC: 0.990).

### Test–retest metrics

**Figure 2.**
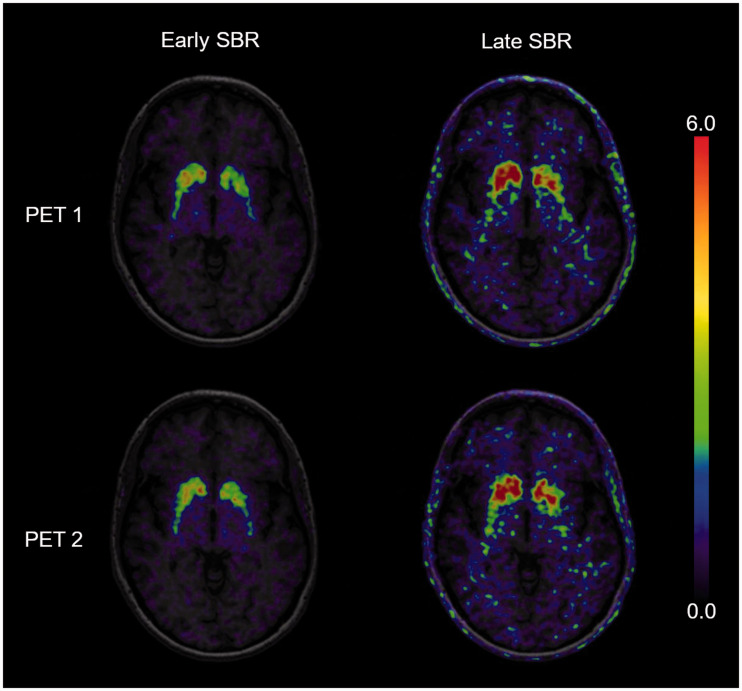
Specific binding ratios (SBR) during the early peak (left) and the late pseudo-equilibrium (right) of one patient, performing two [^18^F]-FEPE2I PET scans within seven days. The cerebral uptake is scaled to the cerebellum with subtraction of the unspecific binding in the reference region.

Test–retest agreement and reliability of binding estimates were overall slightly better for early SBR measures as compared to late SBR measures (Table 3 and Figure 2). The average AbsVar was similar for *BP*_ND_ and early SBR in caudate and putamen (6.6%–7.7%), slightly higher for short early SBR, but clearly higher for late SBR measures (9.4%–15.2%). ICCs were high for all five measures in the caudate and for *BP*_ND_ and late SBR measures in the putamen (≥ 0.90). Early SBR measures showed a lower but still good ICC in the putamen (0.89 and 0.85, respectively). SEM of *BP*_ND_ and early SBR were within the same range in both VOIs (0.05–0.10), slightly lower than for short early SBR (0.15 and 0.11) and clearly lower than for late SBR measures (0.17–0.20).

### Longitudinal DAT binding changes

**Figure 3.**
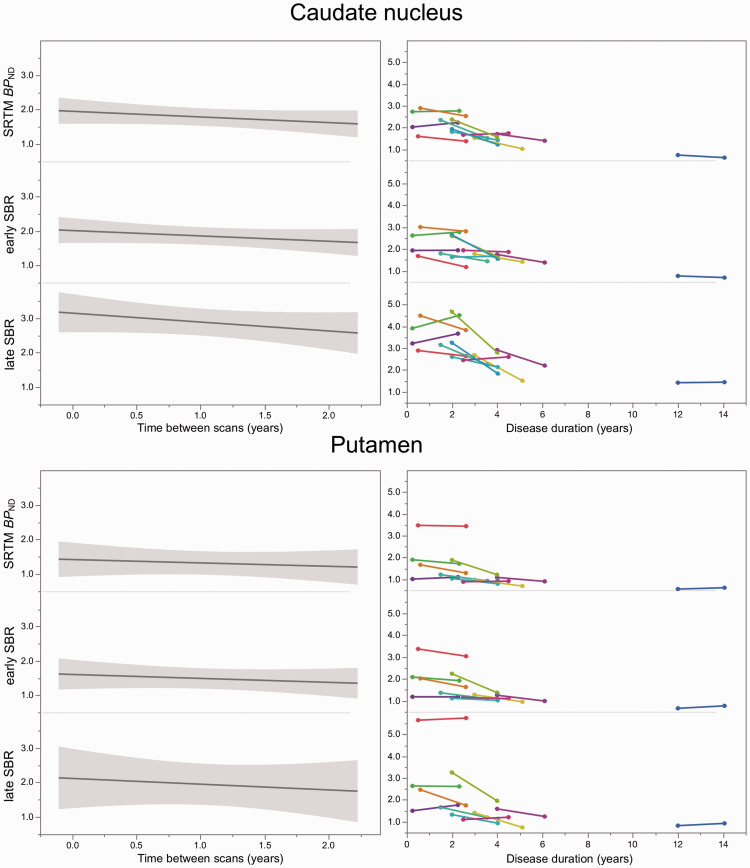
Slope of dopamine transporter (DAT) binding estimates between baseline and two-year follow-up (left panels; with grey shaded 95% confidence intervals). Right panels show the longitudinal changes of the individuals’ DAT binding estimates plotted over disease duration. SRTM: simplified reference tissue model; *BP*_ND_: binding potential; SBR: specific binding ratio.

The average APC was in a narrow range (−7.2% – 8.5%) and almost equal for all binding measures in both striatal regions (Table 3). However, when comparing the DAT binding after two-year follow-up with baseline values, significant within-group differences were observed for *BP*_ND_ (caudate: 1.95 ± 0.58 vs. 1.62 ± 0.62, *p* = 0.01; putamen: 1.44 ± 0.77 vs. 1.21 ± 0.76, *p* = 0.02), early SBR (caudate: 2.01 ± 0.61 vs. 1.70 vs. 0.60, *p* = 0.03; putamen: 1.66 ± 0.75 vs. 1.37 ± 0.61, *p* = 0.02), and short early SBR (caudate: 2.66 ± 0.80 vs. 2.21 ± 0.78, *p* = 0.04; putamen: 2.08 ± 1.02 vs. 1.67 ± 0.84, *p* = 0.01), but not for late SBR (caudate: 3.14 ± 0.90 vs. 2.62 ± 0.95, *p* = 0.07; putamen: 2.15 ± 1.31 vs. 1.77 ± 1.36, *p* = 0.06) and short late SBR (caudate: 3.17 ± 0.91 vs. 2.62 ± 0.96, *p* = 0.05; putamen 2.16 ± 1.33 vs. 1.76 ± 1.37, *p* = 0.06) (Figure 3).

## Discussion

The aim of this study was to directly compare the performance of the simplified quantification of [^18^F]-FE-PE2I PET during early peak and during late pseudo-equilibrium in the context of potential future indications in clinical routine. There are two main findings of this study: first, the SBRs of both equilibrium states showed similar and high discriminative values to differentiate PD patients from controls; second, SBR during the early equilibrium provided less variability and a more favourable reliability than late SBR, suggesting that early SBR should be chosen for simplified DAT quantification.

### Correlation, bias, effect size and discriminative power

Correlational analyses with the *BP*_ND_ showed negligible differences between early and late SBR. The biases associated with the late SBR windows were much larger than the one observed for early SBR, confirming previous findings in larger cohorts of patients and controls.^19,21^ However, late SBR windows tended to show a more uniform bias across regions and groups as compared with early SBR windows. Group differences of all binding estimates in both caudate and putamen reflect the potential of [^18^F]-FE-PE2I PET to separate PD patients from controls. SBR for early windows showed slightly higher effect sizes and lower COV values than SBR for late windows, indicating lower between-subject variability during the early equilibrium. Despite this, ROC analysis in the putamen showed AUC values close to 1 for all five outcome measures. Since the putamen is the region showing the largest dopaminergic depletion,^1^ it can be expected that similar discriminative power can be achieved independently on the accuracy of the quantification. Therefore, all early and late SBRs are appropriate for differential diagnosis in patients with parkinsonism.

Slight differences were found in comparison to previous results. One recent study showed an AUC of only 0.89 for the SBR of [^18^F]-FE-PE2I during the late equilibrium.^20^ The authors attributed this to the fact that the clinical diagnosis of PD was not confirmed at follow-up in four of the patients enrolled as PD, causing normal DAT binding values in the PD cohort. Patients that fulfil the clinical criteria of PD, but show normal DAT binding in the putamen are often referred to as subjects with a scan without evidence of dopaminergic deficit (SWEDD) and are unlikely to maintain the diagnosis of PD after long-term follow-up.^30^ In our study, only one patient (who also participated in the longitudinal study) belongs to this group. The diagnostic accuracy may, therefore, differ between study cohorts. However, all evaluated SBRs agreed with the reference *BP*_ND_ by identifying the SWEDD patient as outlier in the PD cohort, which also supports the suitability of [^18^F]-FE-PE2I PET for patient selection in clinical trials.^12^

### Test–retest metrics and longitudinal analysis

The precise and reliable quantification of DAT binding is of utmost importance for within-subject comparison. In this study, although the ICC was similar for *BP*_ND_, early and late SBR, differences in test–retest metrics between early and late SBR were observed. The average AbsVar of SBR for late windows was much larger than the AbsVar of SBR for early windows and the AbsVar of *BP*_ND_. In addition, the larger SEM during the late equilibrium indicates poorer reliability than during the early equilibrium. The loss of reliability for ratio methods during the late pseudo-equilibrium might be related to the sensitivity of late ratios to the radioligand’s clearance rate from the reference tissue, ^31^ which might be susceptible to inter- and intraindividual variability. The impact of a reduced reliability was also observed in the longitudinal cohort. The annual change of all five outcome measures in our cohort was in agreement with the previously reported range of 5–13% for striatal DAT decline.^32–36^ However, a significant difference between baseline and follow-up was only observed for *BP*_ND_, early SBR, and short early SBR. In the case of longitudinal studies or clinical trials, the higher SEM of SBR for late windows would require a longer interval between baseline and follow-up measurements, or a larger study cohort.

In view of the need to accelerate drug discovery and development for the treatment of alpha-synucleinopathies,^37,38^ the reduction of sample size and study duration is of great relevance to reduce costs and resources. Methodological advancements support this aim, but do not overcome general shortcomings of DAT imaging to measure PD progression and severity, e.g. the flooring effect of DAT availability at advanced disease stages, or the involvement of several brain regions and neurotransmitter systems to PD pathology.^39^ Likewise, possible subtle effects of dopaminergic treatment on DAT binding cannot be excluded,^40–42^ particularly in longitudinal evaluations as in our cohort, in which the levodopa equivalent daily dose increased from 238 ± 204 at baseline to 596 ± 391 at follow-up. To best possible control for such effects in this study, all patients suspended antiparkinsonian medication before PET examinations.

### Choice of short imaging protocol

According to the overall results, if a dynamic acquisition >60 min is not feasible, the imaging protocol of [^18^F]-FE-PE2I PET should follow the purpose. The use of late SBR measures suffices for clinical applications in patients with parkinsonism or when DAT-PET is used as enrichment biomarker in clinical trials. On the other hand, an acquisition protocol covering the early equilibrium should be the preferred method if DAT imaging is used as quantitative marker. This might be the case if DAT-PET with [^18^F]-FE-PE2I is used in clinical trials or as research tool for correlation with clinical parameters (e.g. motor scores, neuropsychological measures, cognition and behaviour). For both equilibrium states, a better count statistic due to an acquisition over 30 min provides less variability and a better reliability as compared to a scan duration of 18 min, and therefore should be considered if possible.

### Limitations

This study has some limitations. Although the aim is to support the translation of [^18^F]-FE-PE2I PET into clinical practice, the data were acquired with a research PET system. To simulate a clinical setting, the HRRT image data were smoothed to a resolution comparable to data derived from a clinical PET system. The analysis of these simulated data (see Supplemental material) confirmed our main findings, though additional studies are needed to corroborate the results obtained with the high-resolution system. In particular, the process of smoothing the data to lower the resolution of the reconstructed images produced a decrease in the SBR values. The overestimation of SBR in comparison with the gold standard *BP*_ND_ values was thus mitigated. This effect might result in an overall uniform bias between groups and regions during the late equilibrium. Such bias needs to be further investigated.

## Conclusion

Simplified quantification of [^18^F]-FE-PE2I PET during either early peak or late pseudo-equilibrium and scan time reduction to 18 min retains discriminative power to separate PD patients and healthy controls. Since early SBR shows better reliability, the acquisition during early peak equilibrium is preferable if [^18^F]-FE-PE2I PET is used as quantitative estimate for disease severity and progression.

## Supplemental Material

sj-pdf-1-jcb-10.1177_0271678X20958755 - Supplemental material for Simplified quantification of [^18^F]FE-PE2I PET in Parkinson’s disease: Discriminative power, test–retest reliability and longitudinal validity during early peak and late pseudo-equilibrium Click here for additional data file. Supplemental material, sj-pdf-1-jcb-10.1177_0271678X20958755 for Simplified quantification of [^18^F]FE-PE2I PET in Parkinson’s disease: Discriminative power, test–retest reliability and longitudinal validity during early peak and late pseudo-equilibrium by Joachim Brumberg, Vera Kerstens, Zsolt Cselényi, Per Svenningsson, Mathias Sundgren, Patrik Fazio and Andrea Varrone in Journal of Cerebral Blood Flow & Metabolism
