# Supplementary material for: Simplified quantification of [18F]FE-PE2I PET in Parkinson’s disease: Discriminative power, test–retest reliability and longitudinal validity during early peak and late pseudo-equilibrium
Source: J Cereb Blood Flow Metab. 2020 Sep 21;41(6):1291–300. doi: 10.1177/0271678X20958755 (PMC8138335; doi:10.1177/0271678X20958755)
Supplement: sj-pdf-1-jcb-10.1177_0271678X20958755 - Supplemental material for Simplified quantification of [18F]FE-PE2I PET in Parkinson’s disease: Discriminative power, test–retest reliability and longitudinal validity during early peak and late pseudo-equilibrium [file sj-pdf-1-jcb-10.1177_0271678X20958755.pdf]

SUPPLEMENTAL DATA

Materials and Methods

Assessment of motor deficits in PD patients

Motor scores were evaluated in clinical medication “on” condition with the Unified Parkinson´s disease Rating scale part III (UPDRS-III) in 19 PD patients (21±6.1), including all patients of the longitudinal cohort (20.4±6.9). Three PD patients (23.7±5.8) were evaluated in “on” state using the version revised by the Movement Disorder Society (MDS-UPDRS-III) [1]. Eleven patients including the test-retest cohort (20.0±9.7) were evaluated in “off” state using the MDS-UPDRS-III (21.5±13.9).

Molar activity, injected radioactivity and injected mass

Molar activity at the time of injection was 87±53 GBq/µmol in controls and 91±90 GBq/µmol in PD patients, and the injected radioactivity was 200±21 MBq and 201±19 MBq in controls and PD patients, respectively. In the subgroups with two PET acquisitions, molar activity and injected radioactivity were as follows: test-retest, 1st scan: 55±17 GBq/µmol / 206±20 MBq, 2nd scan: 101±75 GBq/µmol / 212±22 MBq, longitudinal, 1st scan: 99±60 GBq/µmol / 198±11 MBq, 2nd scan: 44±19 GBq/µmol / 194±18 MBq.

Generation of SUV images with lower resolution

To simulate the conditions of a clinical setting, we smoothed the HRRT images with a 6 mm Gaussian filter to the resolution of an ECAT EXACT HR PET system (Siemens Medical Solutions) as previously described [2]. The data were then used to generate early and late specific binding ratios for 30 min and 18 min time windows based on this lower system resolution (SBR_{HR}), which we considered equivalent to PET data acquired on a clinical PET system.

Results from analysis with data smoothed to lower resolution

Linear regression analysis and dispersion metrics

Regression analysis showed correlations between SRTM BP_{ND} and both, early (r^2 = 0.87, p < 0.001; short early SBR_{HR}: r^2 = 0.89, p < 0.001) and late SBR_{HR} (r^2 = 0.89, p < 0.001; late early SBR_{HR}: r^2 = 0.89, p < 0.001).

Discriminative analysis between PD patients and controls

Two-sample *t* test showed highly significant group differences ($p < 0.0001$) for all measures in both striatal regions. ROC analysis showed excellent capability to differentiate PD patients from controls for both outcome measures in the putamen (early SBR_{HR} AUC: 0.996; short early SBR_{HR} AUC: 0.994; late SBR_{HR} AUC: 0.991; short late SBR_{HR} AUC: 0.991).

Longitudinal DAT binding changes

The comparison of DAT binding after 2-year follow-up with baseline values showed significant group differences for early SBR_{HR} (caudate: 1.51±0.47 vs. 1.28 vs. 0.47, $p = 0.04$. ; putamen: 1.43±0.58 vs. 1.19±0.50, $p = 0.03$), for the putamen of short early SBR_{HR} (caudate: 2.00±0.62 vs. 1.67±0.60, $p = 0.06$; putamen: 1.76±0.78 vs. 1.45±0.68, $p = 0.04$), but not for late SBR_{HR} (caudate: 2.35±0.68 vs. 1.96±0.76, $p = 0.09$; putamen: 1.80±0.97 vs. 1.48±1.04, $p = 0.09$) and short late SBR_{HR} (caudate: 2.36±0.68 vs. 1.96±0.78, $p = 0.07$; putamen: 1.80 vs. 0.98 vs. 1.48±1.05, $p = 0.08$).

Supplemental Table 1. Binding estimates and Percentage Bias in controls and PD patients.

Binding estimates	Controls		PD patients	
	Caudate	Putamen	Caudate	Putamen
SRTM BP_{ND}	2.98±0.66	4.30±0.79	1.73±0.58	1.28±0.56
early SBR_{HR}	2.25±0.55	3.34±0.72	1.41±0.49	1.32±0.49
short early SBR_{HR}	2.99±0.80	4.33±1.08	1.85±0.62	1.60±0.64
late SBR_{HR}	3.74±1.39	5.41±1.85	2.13±0.71	1.62±0.75
short late SBR_{HR}	3.73±1.39	5.41±1.84	2.14±0.71	1.62±0.76

Bias (%)	Controls		PD patients	
	Caudate	Putamen	Caudate	Putamen
early SBR_{HR}	-24.6	-22.2	-18.4	3.2
short early SBR_{HR}	0.41	0.88	6.6	25.6
late SBR_{HR}	25.4	26.0	22.9	27.0
short late SBR_{HR}	25.0	25.9	23.8	27.2

Data are presented as mean±standard deviation; SRTM = Simplified Reference Tissue Model; BP_{ND} = Binding Potential; SBR_{HR} = Specific Binding Ratio.

Supplemental Table 2. Variability, effect size, test-retest metrics and longitudinal change of DAT binding measures.

	Caudate	Putamen	Caudate	Putamen	Caudate	Putamen
	Cohen`s *d*		COV (%)		AbsVar (%)	
SRTM BP_{ND}	1.44	1.84	38.5	64.5	7.4±6.0	6.6±7.6
early SBR_{HR}	1.27	1.73	37.3	53.9	7.4±5.9	8.3±7.3
short early SBR_{HR}	1.27	1.71	38.7	58.2	7.7±4.8	9.2±8.7
late SBR_{HR}	1.23	1.65	46.8	71.6	10.5±9.9	16.2±14.5
short late SBR_{HR}	1.21	1.65	46.4	71.4	10.8±10.6	17.2±15.6
	ICC		SEM		APC (%)	
SRTM BP_{ND}	0.96	0.95	0.10	0.05	-8.3±7.8	-7.3±8.6
early SBR_{HR}	0.97	0.81	0.07	0.07	-7.3±8.1	-7.0±8.6
short early SBR_{HR}	0.95	0.85	0.10	0.11	-7.2±8.9	-7.3±9.4
late SBR_{HR}	0.94	0.85	0.16	0.16	-7.6±11.5	-8.2±13.0
short late SBR_{HR}	0.94	0.84	0.17	0.22	-7.9±11.6	-8.3±13.8

Data are presented as mean±standard deviation; COV = Coefficient of Variation; AbsVar = Absolute Variability; ICC = Intraclass Correlation Coefficient; SEM = Standard Error of Measurement; APC = Annual Percentage Rate of Change; SRTM = Simplified Reference Tissue Model; BP_{ND} = Binding Potential; SBR_{HR} = Specific Binding Ratio.

References

1. Goetz CG, Tilley BC, Shaftman SR, et al. Movement Disorder Society-sponsored revision of the Unified Parkinson's Disease Rating Scale (MDS-UPDRS): scale presentation and clinimetric testing results. *Mov Disord* 2008; 23: 2129-2170.

2. Sonni I, Fazio P, Schain M, et al. Optimal Acquisition Time Window and Simplified Quantification of Dopamine Transporter Availability Using 18F-FE-PE2I in Healthy Controls and Parkinson Disease Patients. *J Nucl Med* 2016; 57: 1529-1534.
